# Involvement of hyaluronan in the adaptive changes of the rat small intestine neuromuscular function after ischemia/reperfusion injury

**DOI:** 10.1038/s41598-020-67876-9

**Published:** 2020-07-13

**Authors:** Michela Bistoletti, Annalisa Bosi, Ilaria Caon, Anna Maria Chiaravalli, Paola Moretto, Angelo Genoni, Elisabetta Moro, Evgenia Karousou, Manuela Viola, Francesca Crema, Andreina Baj, Alberto Passi, Davide Vigetti, Cristina Giaroni

**Affiliations:** 1 Department of Medicine and Surgery, University of Insubria, via H. Dunant 5, Varese, Italy; 2 Department of Pathology, ASST-Sette Laghi, Ospedale di Circolo Viale L. Borri 57, 21100 Varese, Italy; 3 Department of Internal Medicine and Therapeutics, Section of Pharmacology, University of Pavia, Pavia, Italy

**Keywords:** Autonomic nervous system, Glycobiology, Motility disorders

## Abstract

Intestinal ischemia/reperfusion (I/R) injury has severe consequences on myenteric neurons, which can be irreversibly compromised resulting in slowing of transit and hindered food digestion. Myenteric neurons synthesize hyaluronan (HA) to form a well-structured perineuronal net, which undergoes derangement when myenteric ganglia homeostasis is perturbed, i.e. during inflammation. In this study we evaluated HA involvement in rat small intestine myenteric plexus after in vivo I/R injury induced by clamping a branch of the superior mesenteric artery for 60 min, followed by 24 h of reperfusion. In some experiments, 4-methylumbelliferone (4-MU, 25 mg/kg), a HA synthesis inhibitor, was intraperitoneally administered to normal (CTR), sham-operated (SH) and I/R animals for 24 h. In longitudinal muscle myenteric plexus (LMMP) whole-mount preparations, HA binding protein staining as well as HA levels were significantly higher in the I/R group, and were reduced after 4-MU treatment. HA synthase 1 and 2 (HAS1 and HAS2) labelled myenteric neurons and mRNA levels in LMMPs increased in the I/R group with respect to CTR, and were reduced by 4-MU. The efficiency of the gastrointestinal transit was significantly reduced in I/R and 4-MU-treated I/R groups with respect to CTR and SH groups. In the 4-MU-treated I/R group gastric emptying was reduced with respect to the CTR, SH and I/R groups. Carbachol (CCh) and electrical field (EFS, 0.1–40 Hz) stimulated contractions and EFS-induced (10 Hz) NANC relaxations were reduced in the I/R group with respect to both CTR and SH groups. After I/R, 4-MU treatment increased EFS contractions towards control values, but did not affect CCh-induced contractions. NANC on-relaxations after I/R were not influenced by 4-MU treatment. Main alterations in the neurochemical coding of both excitatory (tachykinergic) and inhibitory pathways (iNOS, VIPergic) were also observed after I/R, and were influenced by 4-MU administration. Overall, our data suggest that, after an intestinal I/R damage, changes of HA homeostasis in specific myenteric neuron populations may influence the efficiency of the gastrointestinal transit. We cannot exclude that modulation of HA synthesis in these conditions may ameliorate derangement of the enteric motor function preventing, at least in part, the development of dysmotility.

## Introduction

The gut is one of the most sensitive organs to ischemia–reperfusion (I/R) injury, which may occur in multiple clinical conditions, such as sepsis, shock, trauma, surgery, and is associated with high morbidity and mortality^1^. The initial ischemic insult causes mucosal shedding, barrier dysfunction and bacterial translocation with prolonged reduction of intestinal blood flow^1,2^. The successive reperfusion period, although essential to rescue ischemic tissues, paradoxically initiates a cascade of events that may lead to additional cell injury, exacerbating vascular and tissue damage^3,4^. The I/R insult has severe consequences, not only on the metabolically active intestinal mucosa, but also on other cell types in the enteric microenvironment, including smooth muscle cells, enteric glial cells and neurons. Myenteric neurons are particularly sensitive and can be irreversibly compromised^5^. The consequent neuronal loss may result in slowing of transit and hampered food digestion, suggestive of a long lasting neuropathy^6,7^. The exact mechanisms involved in myenteric plexus alteration after an intestinal I/R injury are largely unknown, although increased inflammatory cell infiltration, pro-inflammatory cytokine release, production of reactive oxygen species (ROS) and increased levels of nitric oxide (NO) are certainly involved^5,8,9^.

We have recently demonstrated that hyaluronan, HA, an unbranched glycosaminoglycan (GAG) component of the extracellular matrix (ECM), may provide an important perineuronal framework within myenteric ganglia, sustaining myenteric neuron homeostasis, similarly to the perineuronal net (PNN) found in the central nervous system (CNS)^10–12^. The PNN is an integral part of some neuronal populations within the CNS and regulates plasticity by controlling communication between neurons, synaptic ion sorting, lateral mobility of protein on the neuronal surface, and protects neurons from oxidative stress and neurotoxins^12,13^. Disruption of the PNN by matrix degrading enzymes is exacerbated by neuroinflammatory conditions and has been suggested to underlay development of neurological and neurodevelopmental disorders^14^. Analogously, during an experimental colitis in rats, in spite of the enhanced HA deposition in the whole intestinal wall, the well-organized HA network was lost, sustaining inflammation-induced myenteric neuron damage^11^. Accordingly, we hypothesize that HA may be mobilized also during an I/R injury in the gut, affecting myenteric neuron function.

HA is synthesized by a family of three transmembrane synthases (HAS1, HAS2, HAS3) on the surface of neuronal plasma membrane, with different molecular weights and at different rates depending on the CNS area and developmental time points^12,15^. HASes are also localized inside the cell (nascent HASes are in the secretory pathway to reach the membrane or HASes are recycled from the membrane)^16,17^. Intriguingly, it is not fully understood whether intracellular HASes are active or not^18^. The biological effects of HA are stringently dependent upon the GAG size, the activation of downstream receptor pathways, and on its chemical modification driven by TNF-stimulated gene-6 (TSG6)^19,20^. Polymers of HA with high molecular weight have anti-inflammatory properties, by recruiting different receptors, such as CD44, toll-like receptors (TLR) 4 and 2 and receptor for HA-mediated motility (RHAMM), towards cell membranes^15,21,22^. However, during pathological states, including chronic inflammation, long HA polymers are cut by hyaluronidases or oxidative stress by ROS into small fragments, which promote immune cell activation and production of pro-inflammatory cytokines, thus favouring an increased inflammatory response^15,23^. In a mouse model of colitis, for example, administration of medium molecular weight exogenous HA was efficacious in relieving inflammation, via TLR4 activation^24,25^, while accumulation of small endogenous HA fragments within the submucosal layer correlated with increased cytokine production, following leukocyte recruitment^26,27^. Seemingly, in the acute stage of stroke injury in man, increased production of low-molecular weight HA in leukocytes in the stroke and peri-infarcted region sustained the associated inflammatory response, while activation of HA-induced cellular signalling in microvessels and neurons had a favourable impact on remodelling processes stimulating angiogenesis, revascularization and survival of susceptible neurons^28^.

In this study, we focused on the impact of HA homeostasis changes in the adaptive responses of myenteric ganglia during an I/R injury in adult rats. This hypothesis was investigated with molecular, morphological and functional approaches. Some experiments were carried out after in vivo treatment with 4-methylumbelliferone (4-MU), which reduces HA production by both depletion of cellular UDP-glucuronic acid necessary for HA synthesis and downregulation of HA synthases^29^.

## Results

### General observations-histological assessment

After occlusion of the terminal branch of the superior mesenteric artery, the corresponding small intestinal segment became purple and returned to a normal pink color after blood flow restoration. Animal recovered uneventfully from anesthesia and once awake were active, ate normally and did not show any sign of distress. A gross visual inspection of the regions subjected to I/R did not reveal major abnormalities with respect to sham-operated and un-operated control animals. Administration of 4-MU, 25 mg/kg, did not induce significant gross morphology changes in intestinal segments obtained from animals subjected to I/R injury, sham-operated and controls, versus the respective untreated groups. In all experimental groups intestinal segments and mesenteric attachments were loser after 4-MU treatment, compared to the untreated groups.

In contrast with other studies, showing the development of mucosal lesions after a transitory occlusion of the superior mesenteric artery in rats for 75 min followed by 24 h of reperfusion^8,30^, in this study, the microscopic evaluation of intestinal sections after I/R did not evidence prominent histological abnormalities in the mucosa and serosal epithelium. This result agrees with histological data obtained by other Authors^31,32^ and may depend both upon the duration of the ischemic injury (60 min vs. 75 min), as well as on the anatomical site of occlusion, i.e. a terminal branch of the superior mesenteric artery in this study, versus the main branch of the superior mesenteric artery, proximal to its origin from the abdominal aorta, leading to a more massive ischemic damage^7,30^ . Signs of cellular suffering were observed in myenteric neurons both in the submucosal and myenteric plexus, displaying swollen soma, cytoplasm vacuolization and ill-defined cellular membrane with respect to control preparations (Supplementary Fig. S1, panels A, C). Nuclear inclusions were sometimes present. The smooth muscle layer was also altered by the I/R injury with cytoplasmic vacuolization and spaces between cells in some regions of *muscularis propria* with respect to control preparations (Supplementary Fig. S1, panels A, C). In sham-operated samples, histological features of neurons or muscle cell suffering were rarely observed. In control and sham-operated groups, 4-MU treatment did not modify the architecture of all intestinal layers as well as of myenteric neurons. In the I/R group, after 4-MU treatment, minimal cytoplasmic vacuolization and spaces were still visible in some regions of the *muscularis propria* and within the myenteric plexus, in addition, myenteric neurons displayed minor signs of cellular suffering with respect to the I/R untreated group. In this latter group no major alterations in the mucosal and submucosal were appreciated (Supplementary Fig. S1, panels C, D).

### Degree of inflammatory damage

In the *muscularis propria* a significant increase in the number of neutrophils per field was observed in the I/R (*P* < 0.0001) with respect to control and sham groups, which was significantly reduced after 4-MU treatment (Supplementary Fig. S1, panels E–G). In the 4-MU-treated I/R group, the number of neutrophils which infiltrate myenteric ganglia was significantly reduced with respect to the I/R untreated group (*P* < 0.05).

In small intestine LMMPs obtained from the sham-operated and I/R groups, the expression of HIF-1α mRNA significantly increased (*P* < 0.05 and *P* < 0.001, respectively) with respect to controls. After 4-MU treatment, HIF-1α mRNA levels were significantly reduced (*P* < 0.05) both in the sham-operated and I/R group, reaching values not significantly different with respect to controls (Supplementary Fig. S1, Panel H).

### 4-MU treatment regulates HA levels in LMMPs after I/R injury

**Figure 1:**
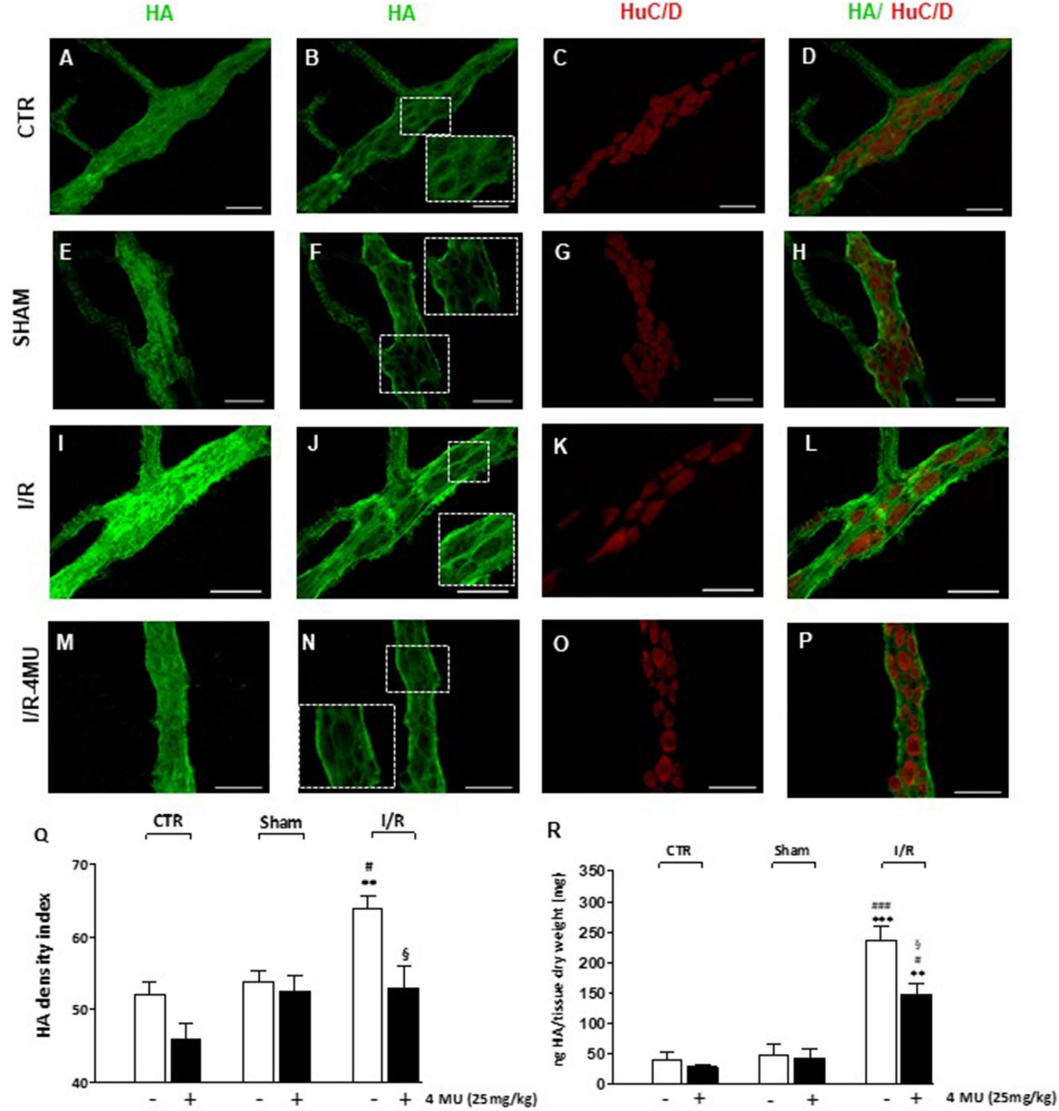
In vivo 4-MU treatment regulates HA levels in rat small intestine LMMPs after I/R injury. (**A–P**) Representative confocal microphotographs showing HA staining of the rat small intestine myenteric plexus in control conditions (CTR, **A**–**D**), in sham-operated animals (**E**–**H**) after ischemia followed by 24 h of reperfusion (I/R, **I**–**L**) and in the I/R group treated with 4-methylumbelliferone (4-MU, 25 mg/kg, ip) (**M**–**P**). In all groups, HA intensely stained the surface of the myenteric ganglia and interconnecting fibers (panels **A**, **E**, **I**, **M**). In myenteric ganglia median sections, HA immunofluorescence was prevalently found in neuronal soma and in the perineuronal space (insets, panels **B**, **F**, **J**, **N**), as demonstrated by double-staining with the pan-neuronal marker, HuC/D. Bar 50 μm. (**Q**) HA density index of HA in LMMP preparations obtained the different experimental groups with and without treatment of 4-MU, as indicated on bottom of bars. Data are reported as mean ± SEM. ^**^P < 0.001 vs. CTR, ^#^*P* < 0.05 vs. sham-operated, ^§^*P* < 0.05 vs. I/R by one-way ANOVA with Tukey’s post hoc test, N = 5 rat/group. (**R**) Quantification of HA levels by ELISA assay in small intestine LMMP preparations obtained from the different experimental groups with and without treatment of 4-MU, as indicated on bottom of bars. HA levels are expressed as ng of HA normalized per mg of dry tissue weight. ***P < 0.001 and **P < 0.01 vs. CTR; ^###^*P* < 0.001 and ^#^*P* < 0.05 vs. sham-operated by one-way ANOVA with Tukey’s post hoc test, N = 6 rat/group.

In LMMP whole-mount preparations from control animals and sham-operated animals, HA staining was detected on the surface of myenteric ganglia and along interconnecting fibers (Fig. 1, panel A, E). Co-localization with the pan-neuronal marker HuC/D demonstrated a faint HA labelling in myenteric neuron cytoplasm and a more intense HA staining in the perineuronal space in preparations obtained from both control and sham-operated animals (Fig. 1, panels B–D, F–H). In both experimental groups, 4-MU treatment did not significantly affect HA labelling in myenteric ganglia (Fig. 1, panel Q). In good agreement, no significant differences of HA levels were detected by ELISA immunoassay in LMMP preparations obtained from control and sham-operated animals, with and without 4-MU treatment (Fig. 1, panel R).

After I/R injury, HA density index significantly increased with respect to control and sham-operated groups (*P* < 0.001 and *P* < 0.05, respectively) as shown in panels I, J and Q of Fig. 1. Accordingly, ELISA assay revealed a significant increase of the GAG levels in LMMP preparations obtained from the I/R group (*P* < 0.01 vs. control and *P* < 0.001 vs. sham-operated) (Fig. 1, panel R). After I/R injury, HA staining in myenteric neuron cytoplasm and in perineuronal space displayed higher intensity with respect to controls and sham-operated groups (Fig. 1, panels I–L), which was reduced by 4-MU treatment (Fig. 1, panels M–P). In the 4-MU treated I/R group, HA levels remained significantly elevated compared to controls and sham-operated groups, but were reduced with respect to the untreated I/R group (Fig. 1, panel R).

### Influence of 4-MU treatment on I/R-induced changes of HAS1 and HAS2 expression in rat small intestine myenteric plexus

**Figure 2:**
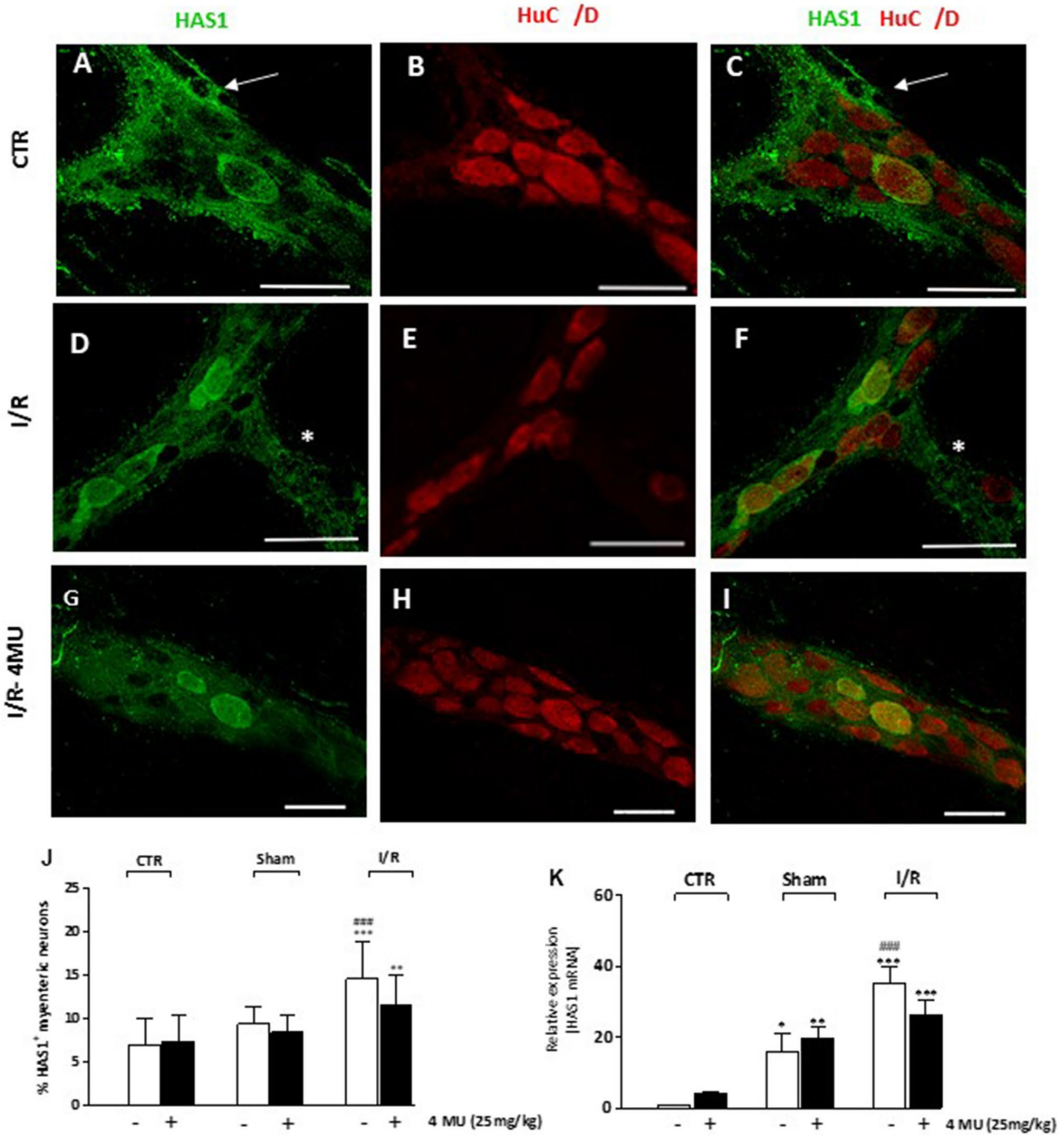
I/R-induced changes of HAS1 expression in the rat small intestine myenteric plexus (**A–I**) Confocal images showing co-localization of HAS1 with the pan neuronal marker HuC/D in myenteric neurons of CTR animals (**A–C**), after I/R injury (**D–F**) and in the 4-MU-treated I/R group (**G–I**). HAS1 stained the soma and cytoplasmic membranes of ovoid myenteric neurons, interconnecting fibers (*) and enteric glial cells (arrow). Panel **J** shows the percentage of myenteric neurons co-staining for HuC/D and HAS1. Bar 50 μm. **P < 0.01, ***P < 0.001 vs. CTR, ^###^*P* < 0.001 vs. sham-operated by one-way ANOVA with Tukey’s post hoc test. N = 5 rat/group (**K**) HAS1 mRNA levels in LMMP preparations obtained from the different experimental groups. Histograms show HAS1 relative gene expression determined by comparing 2^−ΔΔCt^ values normalized to β-actin. *P < 0.05, **P < 0.01, ***P < 0.001 vs. CTR, ^###^*P* < 0.001 vs. sham-operated by one-way ANOVA with Tukey’s post hoc test. N = 5 rat/group.

Immunohistochemical experiments revealed the presence of HAS1 in the cytoplasm and cytosolic membrane of large-, medium- and small-sized ovoid neurons of myenteric ganglia and along interconnecting fibers (Fig. 2, panels A–I). Intense HAS1 labelling was also found in enteric glial cells surrounding myenteric ganglia (Fig. 2, panels A, C). In LMMP preparations obtained from the I/R group, the number of HAS1^+^ myenteric neurons was significantly higher than in control and sham-operated groups (*P* < 0.001) (Fig. 2, panel J). Such enhancement was slightly reduced after 4-MU treatment, remaining, however, significantly higher versus controls (*P* < 0.01) (Fig. 2, panel J). In the sham-operated and I/R groups, HAS1 mRNA levels significantly increased with respect to control preparations (*P* < 0.05 and *P* < 0.001, respectively) (Fig. 2, panel K). In the I/R group, HAS1 mRNA levels were significantly higher than control and in sham-operated animals (*P* < 0.001). After 4-MU treatment, in the sham-operated and I/R groups, HAS1 mRNA levels remained significantly higher with respect to controls (*P* < 0.01 and *P* < 0.001, respectively) (Fig. 2, panel K).

**Figure 3:**
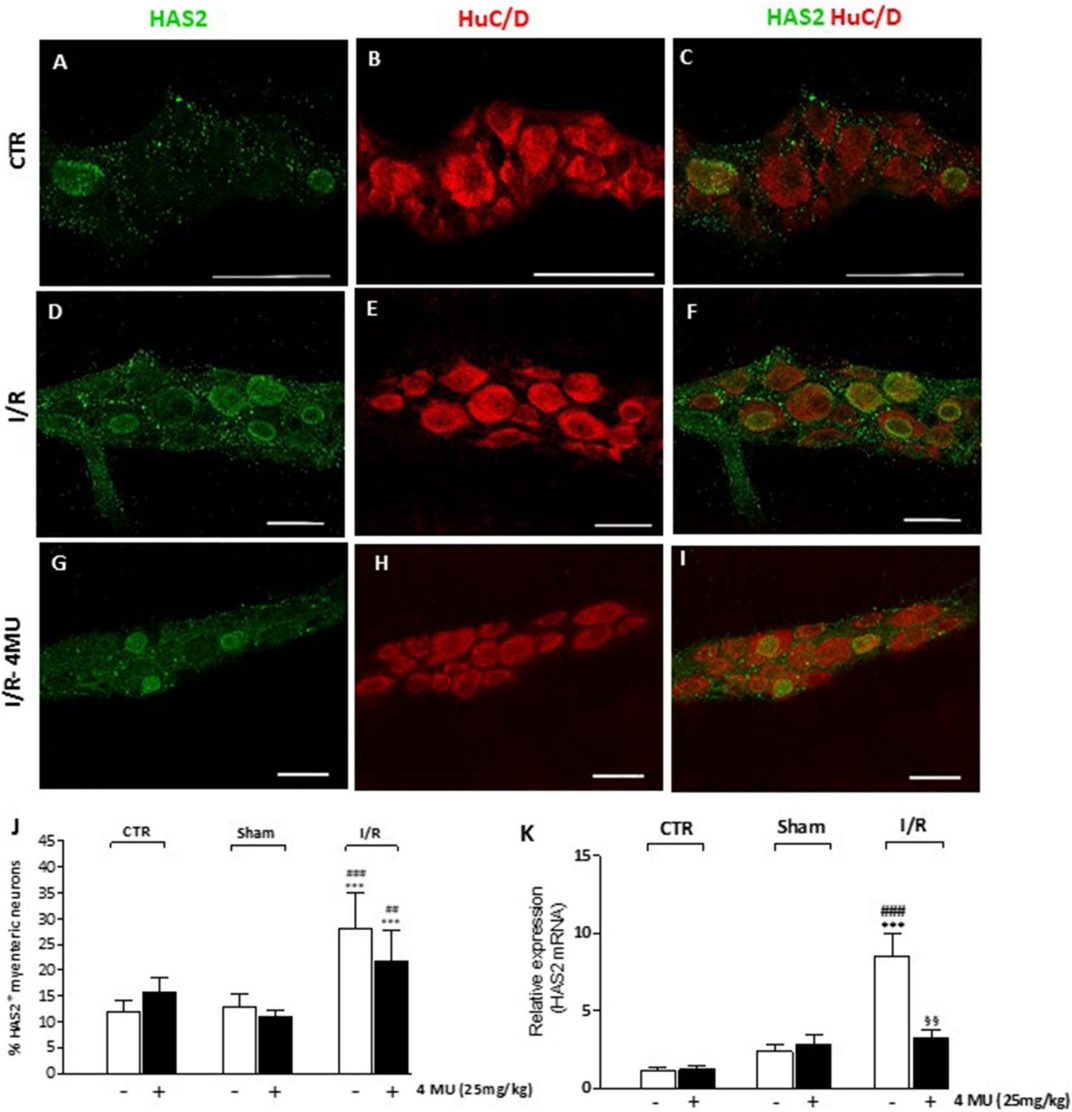
I/R-induced changes of HAS2 expression in the rat small intestine myenteric plexus. (**A–I**) Confocal images showing co-localization of HAS2 with HuC/D in myenteric neurons of CTR animals (**A–C**), after I/R injury (**D–F**) and in the 4-MU-treated I/R group (G-I). HAS2 stained the soma of ovoid myenteric neurons. Panel **J** shows the percentage of myenteric neurons co-staining for HuC/D and HAS2. Bar 50 μm. ***P < 0.001 vs. CTR, ^###^*P* < 0.001 vs. sham-operated by one-way ANOVA with Tukey’s post hoc test. N = 5 rat/group. (**K**) mRNA levels of HAS2 in LMMP preparations obtained from the different experimental groups. Histograms show HAS2 relative gene expression determined by comparing 2^−ΔΔCt^ values normalized to β-actin. ***P < 0.001 vs. CTR, ^###^*P* < 0.001 vs. sham-operated, ^§§^*P* < 0,01 vs. I/R by one-way ANOVA with Tukey’s post hoc test. N = 5 rat/group.

HAS2 immunoreactivity was immunohistochemically found in the cytoplasm, nuclear and cytosolic membranes of few large and medium-sized ovoid myenteric neurons (Fig. 3, panels A–I). The number of HAS2^+^ myenteric neurons significantly (*P* < 0.001) increased with respect to control and sham-operated animals after I/R (Fig. 3, panels D–F, J). In this latter condition, 4-MU treatment induced a tendency towards a reduction of HAS2^+^ myenteric neuron number, which, however, remained significantly higher than in control and sham-operated animals (*P* < 0.001 and *P* < 0.01, respectively) (Fig. 3, panel J). In LMMP preparations obtained from the I/R group, HAS2 mRNA levels significantly increased with respect to both control and sham-operated groups (*P* < 0.001) (Fig. 3, panel K). Exclusively in the I/R group, 4-MU treatment significantly reduced (*P* < 0.01) the enhancement of HAS2 transcript levels with respect to untreated animals, reaching values not significantly different from those obtained in control and sham-operated rats (Fig. 3, panel K). In the control group, the relative expression of HAS1 and HAS2 was not significantly different (2^−ΔΔct^ relative to βactin: 2.80 ± 061, n = 5; 1.87 ± 0.40, n = 5, respectively *P* > 0.05 by Student’ t test). In LMMPs obtained from all the experimental groups, HAS3 mRNA levels were not measurable by qRT-PCR.

### Efficiency of the gastrointestinal transit after I/R injury with and without 4-MU treatment

**Figure 4:**
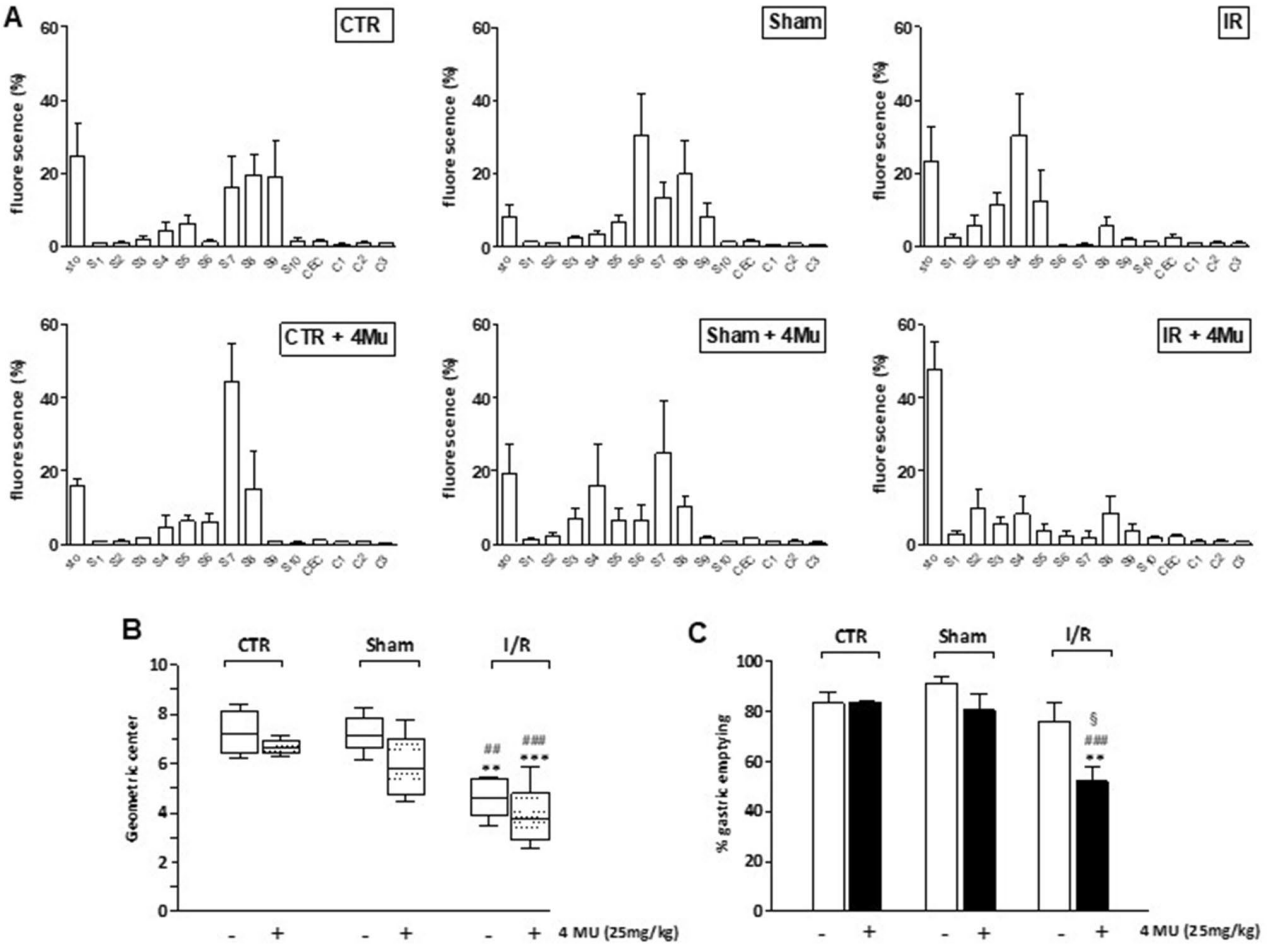
4-MU treatment affects G/I transit and gastric emptying after I/R injury in the rat. (**A**) Percentage of non-absorbable FITC-dextran 250 distribution along the gastrointestinal (GI) consisting of stomach (Sto), small bowel (Sb 1–10), caecum (Cec) and colon (C 1–3); (**B**) geometric centre (GC) of non-absorbable FITC-dextran distribution; (**C**) Percentage of gastric emptying in the different experimental groups. Data are reported as mean ± SEM for panels (**A**) and (**C**) and as median, minimum, maximum, upper and lower quartiles for panel (**B**). **P < 0.01 and ***P < 0.001 vs. CTR, ^##^*P* < 0.01 and ^###^*P* < 0.001 vs. sham-operated, ^§^*P* < 0.05 vs. I/R, by one-way ANOVA with Tukey’s post hoc test (panel **C**). N = 5 rat/group.

After I/R injury, the GI transit significantly decreased, as shown by the higher content of non-absorbable FITC-labelled dextran in the upper part of the GI tract (Fig. 4, panel A). Accordingly, I/R injury induced a significant (*P* < 0.01) reduction of the geometric center (GC) compared with that of control and sham-operated animals. In the I/R group, treatment with 4-MU, induced a significant reduction of the GC (*P* < 0.0001), as well as a significant delay in gastric emptying (*P* < 0.01 and *P* < 0.001, *P* < 0.05), with respect to control, sham-operated and I/R treated animals (Fig. 4, panel B,C). Sham operation, with and without 4-MU treatment, did not significantly influence both the GC and gastric emptying,

### I/R injury alters excitatory neuromuscular contractility: effect of 4-MU treatment

**Figure 5:**
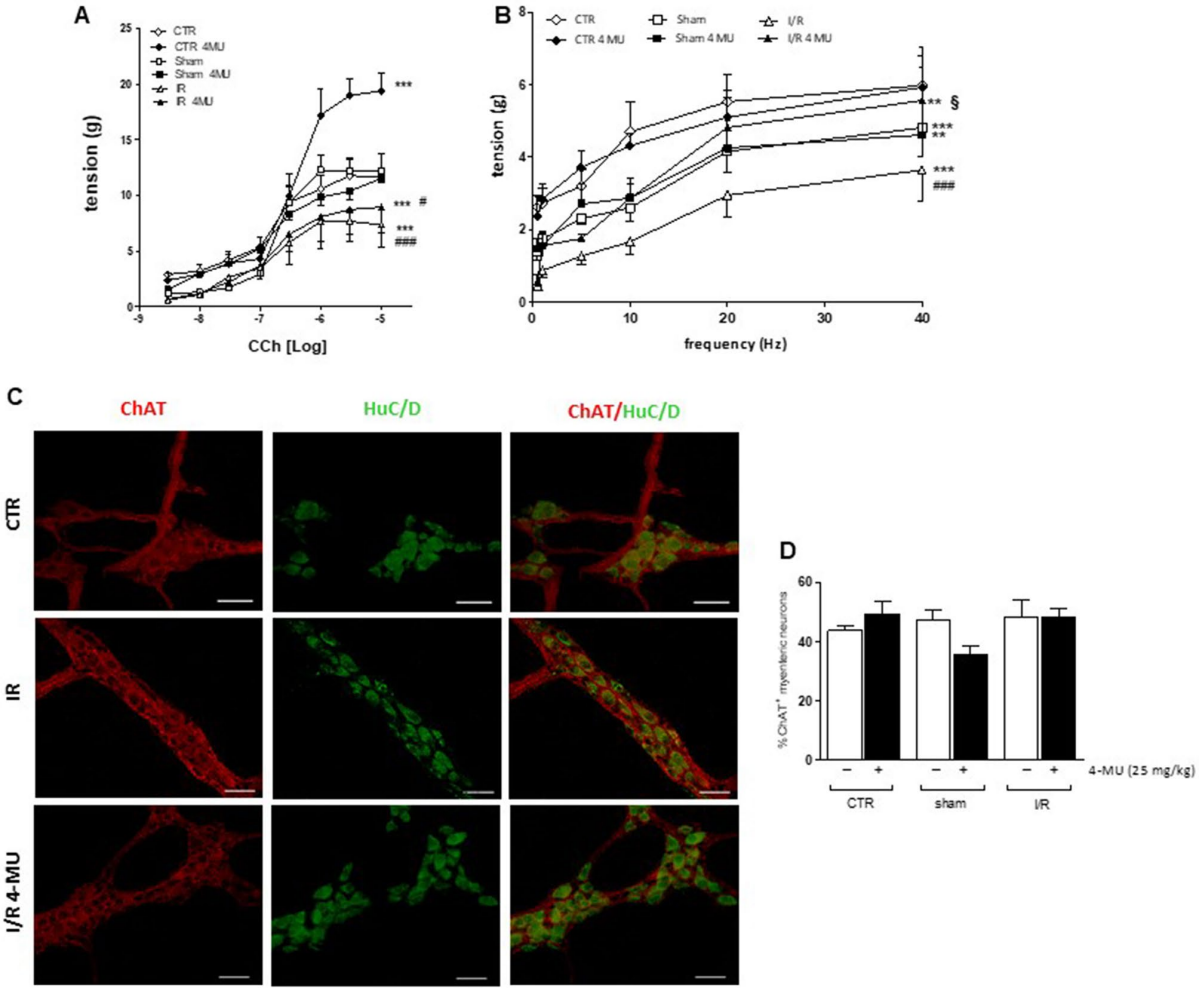
4-MU treatment influences excitatory contractility in the rat small intestine. (**A**) Concentration–response curves to carbachol (CCh) of isolated rat small intestine segments in the different experimental groups (N = 5 rats per group). (**B**) Neuromuscular excitatory responses induced by EFS (0.5–40 Hz) in isolated small intestine preparations obtained from the different experimental groups N = 5 rat/group. (**C**) Representative confocal photomicrographs showing the distribution of ChAT (red, marker for cholinergic neurons) and HuC/D (green, pan-neuronal marker) and (**D**) ChAT density index in LMMP preparations obtained from all experimental groups (N = 5 rat/group). Data are reported as mean ± SEM. (**A**, **B**) statistical significance: **P < 0.01, ***P < 0.001 vs. CTR; ^#^*P* < 0.05 ^###^*P* < 0.001 vs. sham-operated; ^§^*P* < 0.05 vs. I/R by Two- way ANOVA (**A**, **B**).

The effects of I/R injury on the rat small intestine neuromuscular function were examined in vitro by measuring the excitatory contractile response of the longitudinal muscle, which underlies the preparative phase of the peristaltic reflex and is synchronous with circular muscle contraction during peristalsis, thus influencing the whole propulsive bowel activity^33^. In all experimental groups, intestinal specimens showed spontaneous contractile activity consisting of phasic contractions, with significantly reduced amplitude (tension) in the sham-operated and I/R groups with respect to control animals (*P* < 0.01 and *P* < 0.05, respectively), which were not modified by the 4-MU treatment (Supplementary Table S1). The frequency of spontaneous basal contraction was similar in all experimental groups (Supplementary Table S1). To evaluate changes in the excitatory neuromuscular response, cumulative concentration–response curves to the non-selective cholinergic agonist, carbachol (CCh), were performed on longitudinally oriented small intestine segments from all experimental groups. I/R induced a significant downward shift of the concentration–response curve to CCh with a decrease in maximum response (Emax) with respect to values obtained in control and sham-operated animals (*P* < 0.001). (Fig. 5, panel A). In the control group, 4-MU treatment induced a significant increase in the Emax with respect to the value obtained in the respective untreated group (Fig. 5, panel A). In both the sham-operated and I/R group, concentration–response curves to CCh were not significantly modified after 4-MU treatment (Fig. 5, panel A). The potency of CCh in inducing concentration-dependent contractile responses in the control group was 0.17 (0.10–0.28) µM, n = 5, and was not significantly different from those observed in the other experimental groups [CTR-4MU: 0.34 (0.25–0.46) µM, n = 5; sham-operated: 0.20 (0.15–0.27) µM, n = 5; sham-operated-4MU: 0.15 (0.11–0.20) µM, n = 5; I/R: 0.11 (0.036–0.32) µM, n = 5; I/R-4MU: 0.15 (0.06–0.36) µM, n = 5]. To further investigate potential changes in the excitatory contractile function, the effect of EFS was evaluated at increasing frequencies of stimulation on the small intestine longitudinal muscle. In the sham-operated and I/R groups, EFS-elicited contractions were significantly reduced with respect to controls (*P* < 0.001; Fig. 5, panel B). In the control and sham-operated groups, 4MU treatment did not modify the responses to EFS compared to those observed in the respective untreated groups. EFS-induced contractions in the 4-MU treated sham-operated group were significantly lower than those observed in the control group (*P* < 0.01). In the I/R group, after 4-MU treatment, EFS-evoked responses of the small intestine longitudinal muscle were higher versus those obtained in the untreated I/R group (*P* < 0.05), but lower with respect to those obtained in the control group (Fig. 5, panel B). EFS-mediated responses were of neuronal origin, since in all groups addition of the neuronal blocker TTX (1 µM) totally abolished EFS-induced responses. In small intestine myenteric plexus, distribution of ChAT immunoreactivity was similar in all experimental groups as demonstrated by the unchanged density index (Fig. 5, panels C, D).

### I/R injury influences NANC small intestine non-cholinergic excitatory neurotransmission: effect of 4-MU treatment

**Figure 6:**
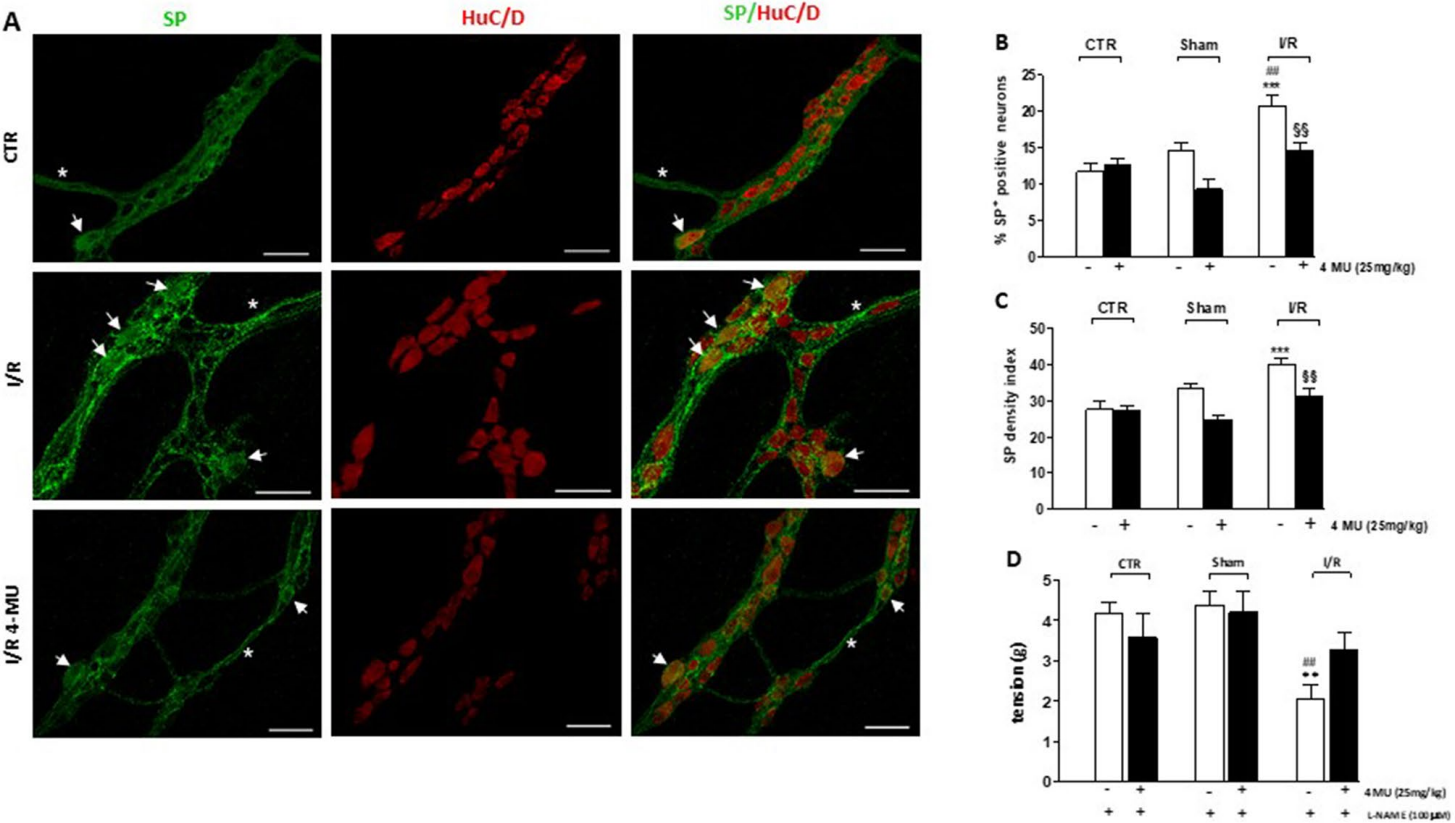
4-MU modulation of tackykinergic neurotransmission in the rat small intestine after I/R injury. (**A**) Representative confocal microphotographs showing the distribution of SP (green) and HuC/D (red) in rat myenteric plexus obtained from CTR, I/R and I/R-4MU groups (N = 5 rats per group). Scale bars = 50 μm. Arrows indicate SP^+^-HuC/D^+^ neurons, asterisk interconnecting fibers. (**B**) Percentage of SP^+^-HuC/D^+^ neurons with respect to total HuC/D^+^ neurons and (**C**) density index of SP immunoreactivity in small intestine LMMP whole-mount preparations in the different experimental groups (N = 5 rats per group). (**D**) Tachykininergic-mediated contractions evoked by 10 Hz EFS in small intestine segments in the different experimental groups (N = 5 rats per group), under NANC conditions, in the presence of L-NAME. Data are reported as mean ± SEM. Difference significance: **P < 0.01, ***P < 0.001 vs. CTR; ^##^*P* < 0.01 vs. sham-operated; ^§§^*P* < 0.01 vs. I/R by one way ANOVA.

In small intestine whole-mount preparations obtained from all experimental groups, immunoreactivity to SP, a neuropeptide of the tachykinin family, was generally faint in the soma of myenteric neurons and more intense in nerve varicose fibers within myenteric ganglia and in interconnecting trunks along the longitudinal muscle (Fig. 6, panel A). In control and sham-operated preparations, with or without 4-MU treatment, the percentage of SP^+^ myenteric neurons and the relevant density index were not significantly different (Fig. 6, panels B, C). In the I/R group, the number of SP^+^ myenteric neurons as well as the density index significantly increased and both effects were significantly reduced by 4-MU treatment (Fig. 6, panels B, C).

To assess the contribution of non-cholinergic excitatory neurotransmitters to the small intestine motor function, post-stimulus excitatory off-responses were evaluated under NANC conditions in the presence of L-NAME, to unmask tachykinergic nerve-evoked contractions^34^. Non-adrenergic non-cholinergic (NANC) responses induced by 10 Hz transmural stimulation were evaluated in the small intestine of all groups in the presence of guanethidine and atropine. Tachykinin-mediated contractions were not significantly different in all groups (Fig. 6, panel D), except for the I/R group in which non-cholinergic excitatory responses were significantly reduced with respect to both control and sham-operated animals and returned to control values after 4-MU treatment (Fig. 6, panel D).

### Effect of 4-MU treatment on the inhibitory neuromuscular response after I/R injury

**Figure 7:**
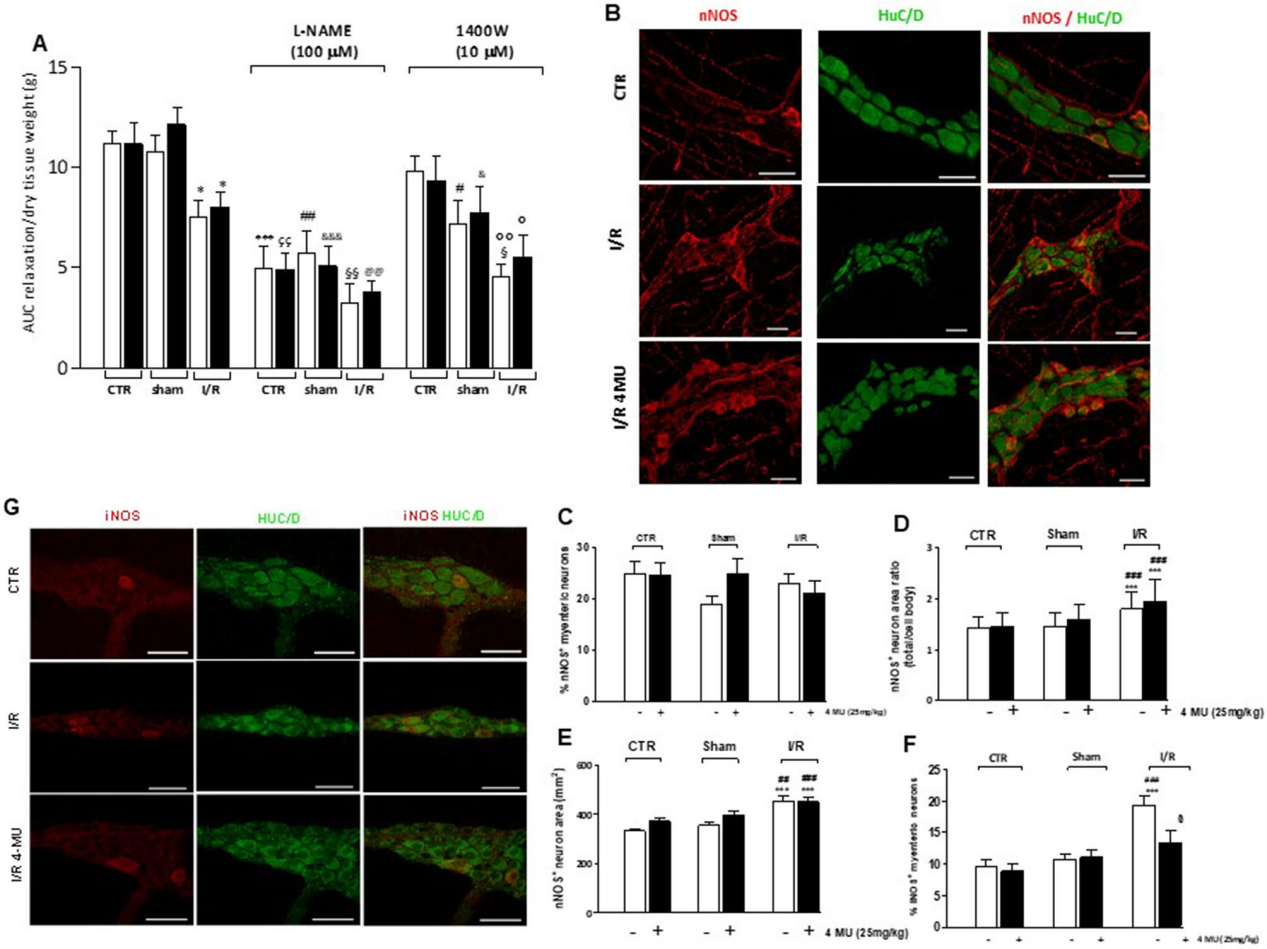
Effect of 4-MU treatment on inhibitory neurotransmission in the rat small intestine. (**A**) 10 Hz EFS-evoked NANC on-relaxation responses of the rat small intestine longitudinal muscle in CTR, sham-operated and I/R (blank columns) and in the same groups after 4-MU treatment (black columns) in the absence and presence L-NAME and 1400 W (N = 5 rats per group). Data are reported as mean ± SEM. Statistical significance: *P < 0.05, ***P < 0.001 vs. CTR; ^ç^*P* < 0.05, ^çç^*P* < 0.01 vs. CTR 4-MU; ^##^*P* < 0.01 vs. sham-operated; ^&&^*P* < 0.01 vs. sham-operated 4-MU; ^§§^*P* < 0.01 vs. I/R; ^@^*P* < 0.05 vs. I/R 4-MU; °*P* < 0.05 and °°*P* < 0.01 vs. 1400 W-treated CTR, by one-way ANOVA followed by Tuckey’s post hoc test. (**B)** Representative confocal photomicrographs showing the distribution of nNOS immunoreactive myenteric neurons (red) and their co-localization with pan neuronal marker HuC/D (green). (**C**) percentage of HuC/D^+^-nNOS^+^ neurons with respect to total HuC/D^+^ neurons in small intestine LMMP whole-mount preparations in the different experimental groups, (N = 5 rats per group). (**D,E**) Morphological analyses of nNOS immunoreactive myenteric neurons in rat small intestine of the different experimental groups. (**D**) Ratios of total cell areas, including dendrites, to cell body area and (**E**) nNOS^+^ cell profile total areas, including dendrites. Values are expressed as means ± SEM. ***P < 0.001 vs. control, ^##^*P* < 0.01 and ^###^*P* < 0.001 vs. sham-operated by one way ANOVA followed by Tukey’s test. (**F)** Percentage of HuC/D^+^-iNOS^+^ neurons with respect to total HuC/D^+^ neurons in ileal LMMP whole-mount preparations in the different experimental groups, (N = 5 rats per group). Values are expressed as means ± SEM. ***P < 0.001 vs. control, ^###^*P* < 0.001 vs. sham-operated and ^§^*P* < 0.05 vs. I/R by one-way ANOVA followed by Tukey’s test. (**G**) Representative confocal photomicrographs showing the distribution of iNOS immunoreactive myenteric neurons (red) and their co-localization with pan neuronal marker HuC/D (green).

In NANC conditions, control and sham-operated segments displayed inhibitory on-relaxation responses of similar amplitude with or without 4-MU treatment (Fig. 7, panel A). In I/R and 4-MU-treated I/R groups, NANC relaxation responses were significantly reduced with respect to control values (*P* < 0.05) (Fig. 7, panel A). In all groups, the inhibitory responses were mediated by NO, as demonstrated by the ability of the non-selective NOS inhibitor, L-NAME, to significantly reduce 10 Hz EFS-induced NANC relaxations with respect to the responses obtained in the absence of the NOS inhibitor (Fig. 7, panel A). In each experimental group, the extent of on-relaxations in the presence of L-NAME was not significantly influenced by 4-MU treatment. In control and 4-MU treated control groups, NANC relaxations were not influenced by pretreatment with 1400 W, a selective iNOS inhibitor (Fig. 7, panel A). In the sham-operated group and 4-MU treated sham-operated group, addition of 1400 W significantly (*P* < 0.05) reduced NANC on-relaxations with respect to values obtained in the absence of the iNOS inhibitor. In the I/R group, 1400 W reduced NANC on-relaxations with respect to the untreated I/R group (*P* < 0.05) and the 1400 W-treated control group (*P* < 0.01), but not versus the 1400 W-treated sham operated group (*P* > 0.05). After 4-MU treatment in the I/R group the reduction of NANC in the presence of 1400 W was not significant with respect to the untreated I/R group (Fig. 7, panel A). In the 4-MU treated I/R group, on-relaxations in the presence of 1400 W were significantly lower than in the 1400 W-treated control group (*P* < 0.05) and did not significantly differ to the values obtained in the 1400 W treated sham-operated group (*P* > 0.05). In each experimental group, the extent of on-relaxations in the presence of 1400 W was not significantly influenced by 4-MU treatment. To further investigate the possible effects of 4-MU treatment on I/R-induced alterations of the enteric nitrergic neurotransmission, we evaluated the distribution of nNOS and iNOS neurons in the myenteric plexus in the different experimental conditions. The number of myenteric neurons per ganglion area, evaluated by staining with the pan neuronal marker HuC/D, was unchanged in the different experimental groups (Supplementary Table S2). Immunohistochemical co-localization with HuC/D showed that the number of nNOS^+^ myenteric neurons was similar in all the experimental groups (Fig. 7, panels B, C). nNOS^+^ neurons displayed morphological changes in both I/R and 4-MU treated I/R groups consisting in a significantly enhanced area/soma ratio and total area with respect to control (*P* < 0.001) and sham-operated groups (*P* < 0.01 and *P* < 0.001) (Fig. 7 panels D, E). In the small intestine of the I/R group, the number of iNOS^+^ neurons significantly increased with respect to control and sham-operated animals (*P* < 0.001). Such enhancement significantly (*P* < 0.05) decreased after 4-MU treatment compared to the untreated group (Fig. 7, panels F, G).

### HA modulates VIPergic neurotransmitter pathways in the rat myenteric plexus after I/R

**Figure 8:**
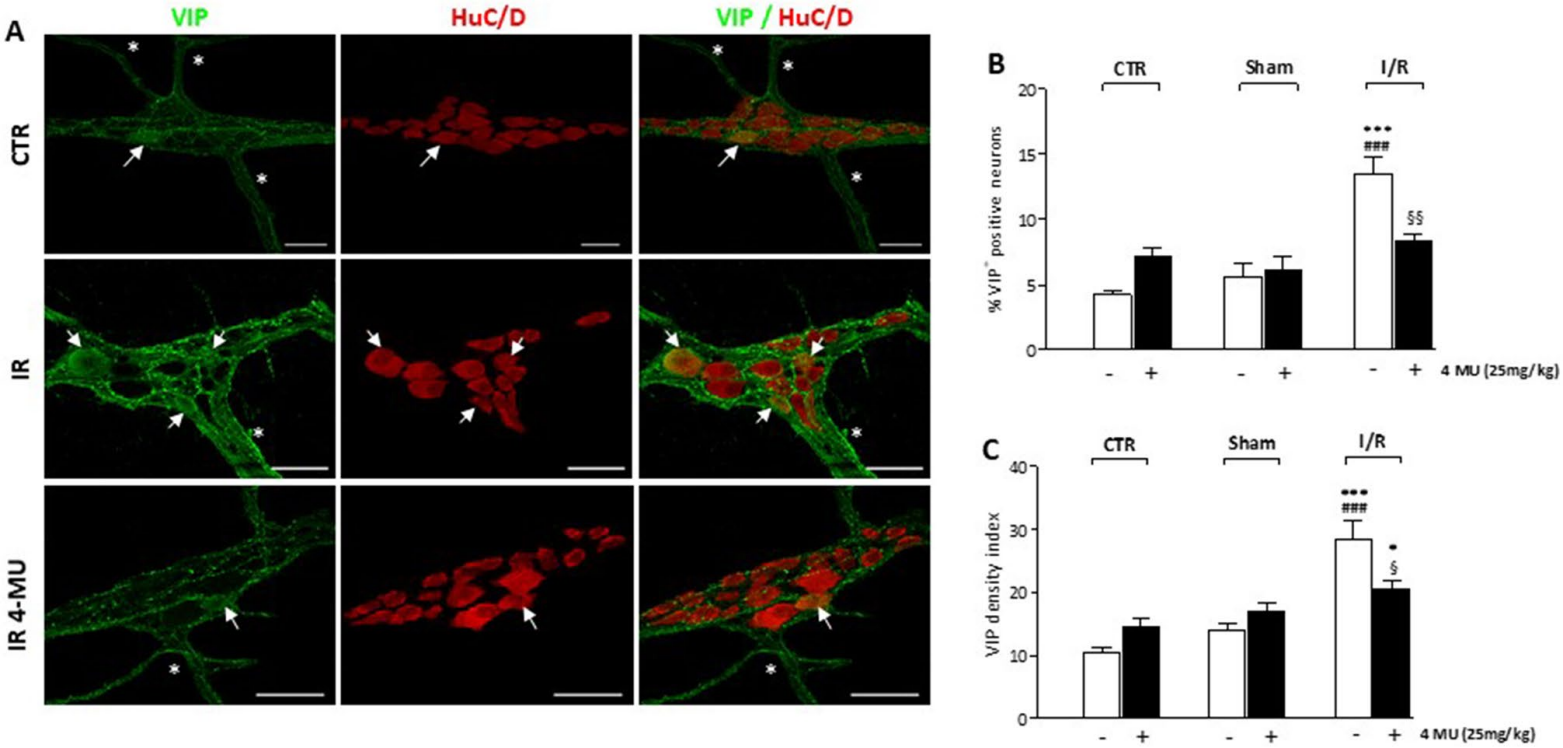
4-MU modulation of VIPergic innervation in the rat small intestine after I/R injury. (**A**) Representative confocal microphotographs showing the distribution of VIP (green) and HuC/D (red) in rat myenteric plexus obtained from CTR, I/R and I/R-4MU groups (N = 5 rats per group). Scale bars = 50 μm. Arrows indicate VIP^+^-HuC/D^+^ neurons, asterisk interconnecting fibers. (**B**) Percentage of VIP^+^-HuC/D^+^ neurons with respect to total HuC/D^+^ neurons and (**C**) density index of VIP immunoreactivity in small intestine LMMP whole-mount preparations in the different experimental groups (N = 5 rats per group). Data are reported as mean ± SEM. Difference significance: **P < 0.01, ***P < 0.001 vs. CTR; ^##^*P* < 0.01 vs. sham-operated; ^§§^*P* < 0.01 vs. I/R by one way ANOVA.

In the ENS, vasoactive intestinal peptide (VIP), acts as an inhibitory neurotransmitter together with NO in the descending reflex of peristalsis and retains fundamental roles in neuronal maintenance and protection during several pathophysiological conditions^35,36^. In small intestine whole-mount preparations obtained from all experimental groups, immunoreactivity to VIP, was generally faint in the soma of myenteric neurons and more intense in nerve varicose fibers within myenteric ganglia and in interconnecting trunks along the longitudinal muscle (Fig. 8, panel A). In control and sham-operated, with and without 4-MU treatment, the percentage of VIP^+^ myenteric neurons and the staining density index were not significantly different (Fig. 8, panels B, C). In the I/R group, the number of VIP^+^ myenteric neurons and the density index significantly increased. Both enhancements were significantly reduced by 4-MU treatment (Fig. 8, panels B, C).

## Discussion

Intestinal I/R injury has detrimental consequences on the ENS structure and function, however, the mechanisms underlying enteric neuron rearrangement in this pathophysiological condition remain largely to be uncovered. In the present study, functional, morphological and biomolecular data show that HA, a glycosaminoglycan (GAG) component of the extracellular matrix (ECM), may influence the neurochemical coding and function of the rat small intestine myenteric neuronal network after an in vivo I/R injury. As recently demonstrated in the rat colon myenteric plexus^11^, HA contributes to the external architecture of rat small intestine myenteric ganglia, by the formation of an ECM coat, in or nearby the basement membrane. In addition, the GAG forms a pericellular coat of condensed matrix surrounding myenteric neurons, similar to the perineuronal net associated with some classes of neurons within the central nervous system (CNS), which is considered fundamental for the regulation of synaptic activity and neuronal plasticity^13^. After I/R, HA levels within the small intestine *muscularis propria* significantly increased with respect to both controls and sham-operated animals, and this change was associated with an increased deposition of the GAG both on ganglia surface and in the perineuronal space. Overall, these observations are in good agreement with previous reports on the accumulation of HA during I/R episodes both in peripheral tissues and in the CNS, suggesting that the GAG may have a role in the pathophysiology of I/R injury^28,37^. As observed in both post mortem human brain and mouse brain after an ischemic stroke^28,38^, the I/R-induced deposition of HA in the rat small intestine myenteric plexus may depend upon up-regulation of HA synthases. The increased number of HAS1^+^ and HAS2^+^ myenteric neurons and the enhancement of mRNA levels of both synthases in longitudinal muscle myenteric plexus (LMMP) preparations, support this hypothesis. HAS1 mRNA, but not the number of HAS1^+^ myenteric neurons, increased also in LMMP preparations obtained from sham-operated animals, possibly owing to the enzyme overexpression in enteric glial cells, in line with the mild inflammatory state observed in this experimental group^5^. In vivo treatment with the HA synthesis inhibitor, 4-methylumbelliferone (4-MU), decreased both HA density index and HA levels in the I/R group more effectively than in control and sham-operated groups, suggesting that the I/R-induced de novo synthesis of HA was principally influenced by 4-MU administration. This observation may reflect the ability of 4-MU to downregulate HA synthases in I/R conditions^29^. However, the number of HAS1^+^ and HAS2^+^ myenteric neurons was only slightly reduced in the I/R group after 4-MU treatment with respect to values observed in the I/R group. As regards HAS1, this tendency was reflected by the modest reduction of its mRNA levels in the 4-MU treated I/R group. Opposite, HAS2 mRNA levels were significantly reduced by 4-MU treatment, suggesting a higher sensitivity of this isoform to 4-MU modulation. Indeed, the inhibitory mechanism of 4-MU is complex and involves both a reduction of UDP-glucuronic acid, a cytosolic precursor of HA, due to UDP-glucuronyl transferases^39^, and a not fully understood downregulation of HASes expression^40^. The results obtained after qRT-PCR analyses of HAS1 and HAS2 transcript levels in the I/R 4 MU-treated group would suggest that HAS2 represents the main enzyme responsible for HA accumulation after I/R. This hypothesis is strengthened by the limited enzymatic capability of HAS1, which has a low affinity for its substrates and catalyzes HA synthesis when UDP-sugar precursors accumulate in the cytosol, as occurs in iperglycemic conditions^41,42^. Interestingly, the mRNA of HIF-1α, which is suggested to induce HA synthesis during hypoxia via HAS2 activation, was downregulated after 4-MU treatment, indicating a further molecular mechanism for the drug-induced HA downregulation^43^.

In the rat small intestine myenteric plexus, changes of HA deposition after I/R are associated with alterations of the intestinal neuromuscular function. In agreement with previous reports, the I/R injury induced a significant slowing of the gastrointestinal transit^5,44^. This latter effect persisted after 4-MU treatment, which, in addition, significantly reduced gastric emptying. These observations suggest that the I/R-induced neo-synthesis of HA may sustain gastrointestinal motor responses during I/R injury. Indeed, in the I/R group 4-MU administration was associated with alterations of the myenteric plexus neurochemical coding of both excitatory and inhibitory neurotransmitter pathways as well as with functional changes, mainly involving excitatory responses. Intriguingly, in contrast with transit and gastric emptying impairment, 4-MU treatment apparently exerted a beneficial effect on the intestinal neuromuscular compartment after I/R, as shown by the reduced signs of damage both in some myenteric neurons and within the smooth muscle cell layer as well as by a lower neutrophilic infiltration. Such discrepancy may depend upon the activation of different signaling pathways triggered by HA of different size^45^. In particular, after I/R, an increased fragmentation of HA may have a detrimental effect on the neuromuscular compartment favoring a neuroinflammatory response, however, both high and low molecular weight HA may also favor the survival and function of specific neuronal populations in myenteric ganglia supporting a remodeling process which supports the intestinal transit^28^.

As regards specific neuromuscular responses, data obtained on carbachol-induced contractions are in good agreement with previous studies showing that a transitory occlusion of the superior mesenteric artery followed by 24 h of reperfusion in rats downregulates muscarinic postjunctional contractile responses^8,44^. I/R-induced impairment of pharmacologically-stimulated longitudinal muscle contractions was unchanged after 4-MU treatment, suggesting that changes in HA deposition do not influence postjunctional cholinergic excitatory responses. In control conditions, however, treatment with 4-MU, induced a significant enhancement of the E_max_ in the carbachol concentration–response curve, suggesting that modulation of HA synthesis within the *muscularis propria* may influence muscarinic postjunctional responses, possibly by favoring receptor recruitment, or by enhancing receptor coupling with effector systems in smooth muscle cells.

In agreement with previous data^31^, in the sham-operated group, TTX-sensitive EFS-evoked contractions were significantly lower than in controls and were further significantly depressed after I/R injury, indicating that the neuronal component of the excitatory contractile response was impaired in both conditions. Administration of 4-MU considerably increased the EFS-evoked response in the I/R, but not in sham-operated animals, suggesting that HA may influence the efficiency of excitatory neuronal pathways only in specific pathophysiological conditions, i.e. after I/R injury, and not under conditions of mild inflammation. Several studies suggest that excitatory cholinergic neurons may be particularly vulnerable to hypoxic/ischemic insults and there are evidences that hypoxia may preferentially depress cholinergic neuronal pathways involved in the intestinal reflexes^46,47,48^. In this study, ChAT^+^ density index in myenteric ganglia was unchanged in the I/R and 4-MU treated I/R group with respect to controls, indicating that alterations of the EFS-induced contractions do not depend upon major changes in the cholinergic innervation. However, we cannot exclude the occurrence of alteration in the synaptic turnover of acetylcholine and/or of other excitatory co-transmitters, such as tachykinins or glutamate^46,49^, which undergo adaptive changes in the myenteric plexus in different pathophysiological conditions, including I/R injury^50,51^. Interestingly, in the I/R group, tachykinin-mediated NANC off-contractions were significantly reduced and were enhanced to control values after 4-MU treatment. These data strongly support the hypothesis that, in I/R conditions, inhibition of HA synthesis by 4-MU may enhance the function of excitatory non-cholinergic contractions, which are depressed by I/R injury. I/R-induced depression of NANC off-contractions and the effect of 4-MU are apparently in contrast with the enhancement of SP^+^ myenteric neurons and SP density index observed after I/R as well as by the inhibitory action of 4-MU on both parameters. Our data are in line with several reports suggesting that SP activity and release may increase after severe acute brain injury, including stroke, where the peptide is critically involved in the development of vasogenic edema and neuronal damage^52,53^. Our data on SP expression in myenteric neurons, with and without 4-MU treatment, suggest that HA promotes up-regulation of SP expression in myenteric neurons after an I/R injury. Such enhancement may induce downregulation of postjunctional tachykinin receptors, underlying reduced NANC off-contraction. All these biomolecular and functional effects are reversed by 4-MU treatment and seem to be specifically correlated with the I/R injury since in the sham-operated group, with and without 4-MU treatment, both immunohistochemical and functional responses did not significantly differ from controls.

Several studies suggest that gut I/R injury is associated with impairment of the NANC inhibitory transmission^6,54^. Accordingly, in this study, a significant reduction of NANC on-relaxations was observed after in vivo I/R and was not influenced by 4-MU treatment. In all experimental groups, sensitivity of NANC relaxations to the non-selective nitric oxide inhibitor, L-NAME, suggests that NO represents a major component of the inhibitory response^5,55,56^. In small intestine segments obtained from 4-MU treated and untreated control animals, NANC on-relaxations were insensitive to the iNOS inhibitor, 1400 W, reflecting the low number of constitutively iNOS expressing myenteric neurons in both groups. In 4-MU treated and untreated sham-operated animals, the mild inflammatory state, may account for the enhanced sensitivity to 1400 W. In the I/R group, the significant reduction of the NANC inhibitory response in the presence of 1400 W indicates that iNOS is the main NOS isoform involved, as suggested by the enhancement of iNOS^+^ myenteric neurons^5,54^. Up-regulation of iNOS^+^ neurons was significantly reduced after 4-MU treatment, indicating that blockade of HA synthesis may influence iNOS expression in myenteric neurons, in good agreement with reports on the ability of HA to induce iNOS mRNA^57^. However, since the effect of 1400 W on NANC on-relaxation after I/R was not significantly affected by 4-MU treatment, the decreased expression of iNOS^+^ in myenteric neurons after HA synthesis blockade does not apparently have consequences on the overall activity of the enzyme in the control of the neuromuscular function during I/R. The number of nNOS^+^ myenteric neurons was similar in all experimental group. However, as previously described^5,54^, important morphological alterations were observed in nNOS^+^ neurons, which were maintained after 4-MU treatment, suggesting that during and I/R injury HA deposition influences enteric nitrergic transmission, mainly via iNOS, but not nNOS. In addition, alternative inhibitory neurotransmitter pathways, comprising purines and peptides may sustain the on-relaxation during I/R^36,58^. Interestingly, 4-MU treatment attenuated the I/R induced increase of VIP^+^ neurons and density index, suggesting that HA is fundamental for the upregulation of VIPergic pathways in this pathophysiological condition^35,59^. However, other inhibitory neurotransmitters may take over NO and VIP in the modulation of on-relaxations in the I/R 4-MU treated group, since the extent of the inhibitory response is similar to that observed in the I/R group. In view of the neuroprotective, vasodilatory and antioxidant properties of VIP^35,59^, the ability of HA to sustain the peptide upregulation in myenteric ganglia during I/R further strengthen the hypothesis that the GAG may have a neuroprotective function in our model.

In conclusion, this study suggests that, during an I/R injury, HA sustains the efficiency of the gastrointestinal transit influencing both excitatory and inhibitory components of the peristaltic reflex. In this condition, after blockade of HA synthesis excitatory neuronal pathways, mainly tachykinergic, are up-regulated, while inhibitory responses remain downregulated, overall deteriorating transit efficiency. The beneficial effects of HA may depend upon the ability of the neo-formed GAG to target specific neuronal populations in these pathological conditions.

## Materials and methods

### Animal models

Male Wistar rats (weight 250–350 g, Envigo, Udine, Italy), were housed under controlled environmental conditions (temperature 22 ± 2 °C; relative humidity 60–70%) with free access to a standard laboratory chow and tap water, and were maintained at a regular 12/12-h light/dark cycle. Their care and handling were in accordance with the provision of the European Union Council Directive 2010/63, recognized and adopted by the Italian Government (Decree No. 26/2014). The experimental protocol was approved by the Animal Care and Use Ethics Committee of University of Insubria and by the Italian Ministry of Health (n°415/2016-PR).

### Ischemia/reperfusion injury

Rats were anesthetized with thiopental sodium (50 mg/kg) diluted (2% w/v) in sterile isotonic saline and given intraperitoneally (i.p.), in non-fasted state. Up to two additional doses, consisting of the 10% of the initial dose, were administered, as needed, in order to maintain anesthesia. After laparatomy, a loop of the small intestine was exteriorized and a branch of the superior mesenteric artery (SMA) supplying the segment was temporarily occluded for 60 min with an atraumatic microvascular clamp, as described by Filpa et al.^5^ and were allowed food and water access after recovery from anesthesia. Sham-operated animals, underwent the same surgical manipulation, except for SMA occlusion. Normal un-operated rats were used as controls. Animals were euthanized 24 h after reperfusion, when the major histopathological alterations and gastrointestinal functional changes have been observed^5^. Further experimental groups were represented by I/R, sham-operated and control animals treated with 4-methylumbelliferone (4-MU, 25 mg/kg i.p.) suspended in DMSO/0.9% NaCl (1:4), 24 h before euthanization. The dose and route of administration of 4-MU did not cause any adverse effect on rats. This dosage was chosen considering its efficacy in downregulating HA production, demonstrated by previous studies^60^. At the end of reperfusion period, rats were euthanized by decapitation and segments of the small intestine, 5 cm oral to the ileo-cecal junction, were rapidly excised and rinsed with a physiological ice-cold Krebs solution [composition (mM): 118 NaCl, KCl , 2.5 CaCl_2_∙2H_2_O, 1.2 MgSO_4_∙7H_2_O, 1.2 K_2_HPO_4_, 25 NaHCO_3_, 11 C_6_H_12_O_6_]. Whole-wall intestinal segments were fixed and stored for successive immunohistochemistry experiments. Quantification of HA levels, western immunoblot and qRT-PCR studies were carried using preparations consisting of external longitudinal muscle layer segments with attached myenteric plexus (LMMP) obtained immediately after excision of small intestine segments, and stored at − 80 °C.

### Histology

Full-thickness small intestine samples were fixed in buffered formalin (4% w/v formaldehyde and acetate buffer 0.05 M) for 24–48 h and routinely embedded in paraffin. Three-micrometer-thick sections were stained with haematoxylin–eosin (HE) for morphologic evaluation. Additional sections were mounted on poly-L-lysine-coated slides for immunohistochemical analysis of neutrophil infiltration. Immunohistochemistry was performed with the avidin–biotin–peroxidase method^5^ using a polyclonal antibody anti-MPO (DAKO, Glostrup, Denmark). Endogenous peroxidase activity was blocked by immersing sections for 10 min in a solution of 3% hydrogen peroxide in water and the primary antibody was incubated overnight at 4 °C. Specific biotinylated secondary antibody and avidin–biotin–peroxidase complex were consecutively applied, each for 1 h at room temperature. The immunohistochemical reaction was developed with diaminobenzidine–hydrogen peroxide reaction. Sections were counterstained with haematoxylin. Neutrophil infiltration was evaluated only in whole well oriented sections of intestine, counting MPO^+^ cells in four high power fields (400x, diameter 0.55 mm). MPO value has been reported as the average of MPO^+^ cells for field in each layer.

### Whole-mount immunohistochemistry

Segments of the rat small intestine were fixed in 4% formaldehyde with 0.2% picric acid in 0.2 mol/l phosphate buffer saline [PBS, composition (mM): 140 NaCl, 3 KCl, 15 Na_2_HPO_4_, 15 KH_2_PO_4_, pH 7.4], for 3–4 h at room temperature (RT). Preparations were then cleared of fixative and stored at 4 °C in PBS containing 0.05% thimerosal. LMMP whole-mount preparations were prepared according to the method described by Ceccotti et al.^61^ The immuno-localization of HA was performed, using a biotin-labeled HA-binding protein (HABP, Hokudo Co, Japan), that recognizes HA saccharidic sequences and is able to localize HA in tissues by streptavidin conjugation with an appropriate fluorophore. Double-labeling was performed during consecutive incubation times of the primary and secondary antibodies, whose related optimal dilutions are described in Supplementary Table S3. Preparations were mounted onto glass slides, using a mounting medium with DAPI (Vectashield; Vector Lab, Burlingame, CA, USA). All data were collected from at least 5 animals for each experimental group, employing a cohort size of 10–20 ganglia. Neuron counts were made on HuC/D stained LMMPs and the total value was divided by the total myenteric ganglion area (μm^2^).To establish the proportion of HAS1, HAS2, ChAT, nNOS, iNOS, SP and VIP expressing myenteric neurons, quantitative analysis of double fluorescently labeled small intestine whole-mount preparations was performed as previously described by Bistoletti et al.^62^. Morphological changes of nNOS^+^ neurons were evaluated by measuring both the total neuronal profile area and the ratio obtained from normalizing the total neuronal profile area with the cell body profile area, as described by Filpa et al.^5^. Immunoreactivity for the different targets was also assessed by analyzing the density index of labelling per myenteric ganglia area (10 fields per preparation at 40 × magnification), as described in Caputi et al.^63^. Preparations were analyzed by confocal microscopy on a Leica TCS SP5 confocal laser scanning system (Leica Microsystems GmbH, Wetzlar, Germany) and pictures were processed using Adobe-Photoshop CS6.0 software.

### HA ELISA assay

In LMMP preparations obtained from all groups, levels of HA were evaluated using a Hyaluronan Quantikine ELISA Kit (R&D Systems, Minneapolis, MN), following the manufacturer’s instructions. In brief, frozen LMMPs from 6 rats per group were lyophilized overnight and then suspended in cell lysis buffer 2 overnight at RT, under gentle agitation. Debris were removed by centrifugation and supernatants were collected for ELISA assay. Absorbance (Abs) values were recorded at 450 nm, with correction applied for optical imperfections in the plate performed by subtracting the Abs readings at 570 nm. HA levels were expressed as ng of HA per mg of dry tissue.

### Gastrointestinal transit analysis

Gastrointestinal transit was measured by evaluating the distribution of an intragastric gavage of fluorescein isothiocyanate (FITC)-labeled dextran (250 kDa, FD250; 6.25 mg/ml dissolved in 0.9% saline). Control, I/R and sham operated animals, with and without 4-MU treatment, were euthanized 90 min after FD250 administration and the entire GI tract was carefully removed and divided into 15 segments: a single stomach segment (sto), 10 equal-length segments of small intestine (S1–S10), a single caecum segment (CEC), and 3 equal-length segments of colon (C1–C3). Except for normal 4-MU un-treated animals (controls), all experimental groups underwent FD250 administration 24 h after the surgical and/or pharmacological procedure. Luminal contents from each sample were collected and clarified by centrifugation (12,000 rpm, 10 min). The cleared supernatants were fluorimetrically measured in duplicate for FD250 intensity, at 492/521 nm using a microplate reader (Infinite 200pro, TECAN). Data were expressed as the percentage of fluorescence for each segment with respect to the total fluorescence along the gastrointestinal tract. The efficiency of FITC-dextran transit along the gastrointestinal tract was determined by calculating the geometric center (GC) for the distribution of the fluorescent probe using the following equation: GC = Σ (% of fluorescence signal per segment x segment number)/100^63^.

### Excitatory and inhibitory in vitro motor responses

Segments of the small intestine (1 cm) were rapidly excised 5 cm oral from the ileo-caecal junction, flushed with Krebs solution, cleared of connective tissue and mounted in isolated baths containing 10 ml of continuously oxygenated (95% O_2_ and 5% CO_2_) and heated (32 ± 1 °C) Krebs solution. Silk ligatures were applied to each end of the segment positioned along the longitudinal axis; one end was attached to a rigid support and the other to an isometric force displacement transducer (MDE Research GmbH, Walldorf, GE). Mechanical activity was recorded with a PowerLab acquisition data system 8 (AD Instruments, UK) and elaborated with a LabChart 4.0 program (AD Instruments, UK). An initial load of 1 g was applied to each intestinal specimen. Tissues were allowed to equilibrate for 60 min prior to the start of the experiments. For each segment, concentration–response curves to the muscarinic agonist, carbachol (CCh) were constructed cumulatively and plotted into a nonlinear regression model (fitted to a sigmoidal equation) to calculate EC_50_ and maximal tension (E_max_) values, to evaluate postjunctional cholinergic responses. Neuronally mediated contractions were obtained by Electric Field Stimulation (EFS, 0–40 Hz; 1-ms pulse duration, 10-s pulse train, 40 V) using platinum bipolar co-axial electrodes, attached to a MDE electronic stimulator (MDE Research). Frequency–response curves were repeated in the presence of tetrodotoxin (TTX; 1 µM) to identify if the response was of neuronal origin.

Non-adrenergic non-cholinergic (NANC) responses were measured at a frequency of 10 Hz after an incubation period of 20 min with atropine (1 μM) and guanethidine (1 μM). In order to evaluate the nitrergic inhibitory neurotransmission, small intestine segments were incubated with L-Nω-Nitroarginine methyl ester chloridrate (L-NAME, a non-selective NOS inhibitor, 100 μM), or 1400 W (a selective iNOS inhibitor, 10 μM), under NANC conditions. To evaluate the tachykinergic component of the small intestine contraction, the 10 Hz EFS-mediated off-response was assessed in presence of L-NAME, under NANC conditions. Under NANC conditions, EFS induced a primary on-relaxation of small intestine segments that was calculated as the AUC and normalized per g dry tissue weight to allow comparisons between tissue samples. Contractile off-responses were expressed as g tension/g dry tissue weight of small intestine segments.

### RNA isolation and quantitative RT-PCR

Total RNA from small intestine LMMPs was extracted with TRIzol (Invitrogen) and treated with DNase I (DNase Free, Ambion), to remove possible traces of contaminating DNA. 2.5 μg of total RNA were then retrotranscribed using the High Capacity cDNA Synthesis Kit (Applied Biosystems, ThermoScientific, Massachusetts, USA) as previously described^64^. Quantitative RT-PCR (qRT-PCR) was performed with an Abi Prism 7,000 real-time thermocyclator (Applied Biosystems). TaqMan Gene Expression Mastermix (Applied Biosystems) was used to detect HAS1 (Rn00597231_m1), HAS2 (Rn00565774_m1) and the housekeeping gene β-actin (Rn00667869_m1) mRNA levels, following the manufacturer’s instructions. To evaluate the expression of HIF-1α, standard qRT-PCR was carried out with Power Sybr Green Universal PCR Master Mix (Applied Biosystems), as indicated by manufacturer's instructions. Primers were designed using Primer Express software (Applied Biosystems) on the basis of available sequences deposited in public database. Primer sequences were: HIF-1α fw 5′-AAGCACTAGACAAAGCTCACCTG-3′, rev 5′-TTGACCATATCGCTGTCCAC-3′ and β-actin fw 5′-TGACAGGATGCAGAAGGAGA-3′, rev 5′-TAGAGCCACCAATCCACACA-3′. Primers were designed to have a similar amplicon size and similar amplification efficiency, and were used at a final concentration of 500 nmol/L for each. The 2^−ΔΔCt^ method was applied to compare the relative gene expression. β-actin was used as housekeeping gene. Experiments were performed at least four times for each different preparation.

### Statistical analysis

All results are reported as mean ± standard error of the mean (SEM), except for the geometric center, which is presented as median and range (minimum–maximum), of at least 5 experiments. Statistical significance was calculated by unpaired Student’s t test, one-way ANOVA followed by Tukey’s post-hoc test or by two-way ANOVA for multiple variable, where appropriate. Differences were considered statistically significant when *P* values were < 0.05. For statistical analysis the GraphPad Prism software was used (GraphPad 5.03 Software Inc, La Jolla, USA).

## Supplementary information

Supplementary file1
