# Supplementary material for: Involvement of hyaluronan in the adaptive changes of the rat small intestine neuromuscular function after ischemia/reperfusion injury
Source: Sci Rep. 2020 Jul 13;10:11521. doi: 10.1038/s41598-020-67876-9 (PMC7359366; doi:10.1038/s41598-020-67876-9)
Supplement: Supplementary file 1 — Supplementary file1 [file 41598_2020_67876_MOESM1_ESM.pdf]

Involvement of hyaluronan in the adaptive changes of the rat small intestine neuromuscular function after ischemia/reperfusion injury

Michela Bistoletti[1#], Annalisa Bosi[1#], Ilaria Caon[1#], Anna Maria Chiaravalli[2], Paola Moretto[1], Angelo Genoni[1], Elisabetta Moro[3], Evgenia Karousou[1], Manuela Viola[1], Francesca Crema[3], Andreina Baj[1], Alberto Passi[1], Davide Vigetti[1,*§], Cristina Giaroni[1,*§].

[1]Department of Medicine and Surgery, University of Insubria, via H. Dunant 5, Varese, Italy

[2]Department of Pathology, Ospedale di Circolo, ASST-Sette Laghi, Viale L. Borri 57, 21100 Varese, Italy

[3]Department of Internal Medicine and Therapeutics, Section of Pharmacology, University of Pavia, Pavia, Italy

This PDF file includes:

Figure S1

Table S1

Table S2

Table S3

Figure S1

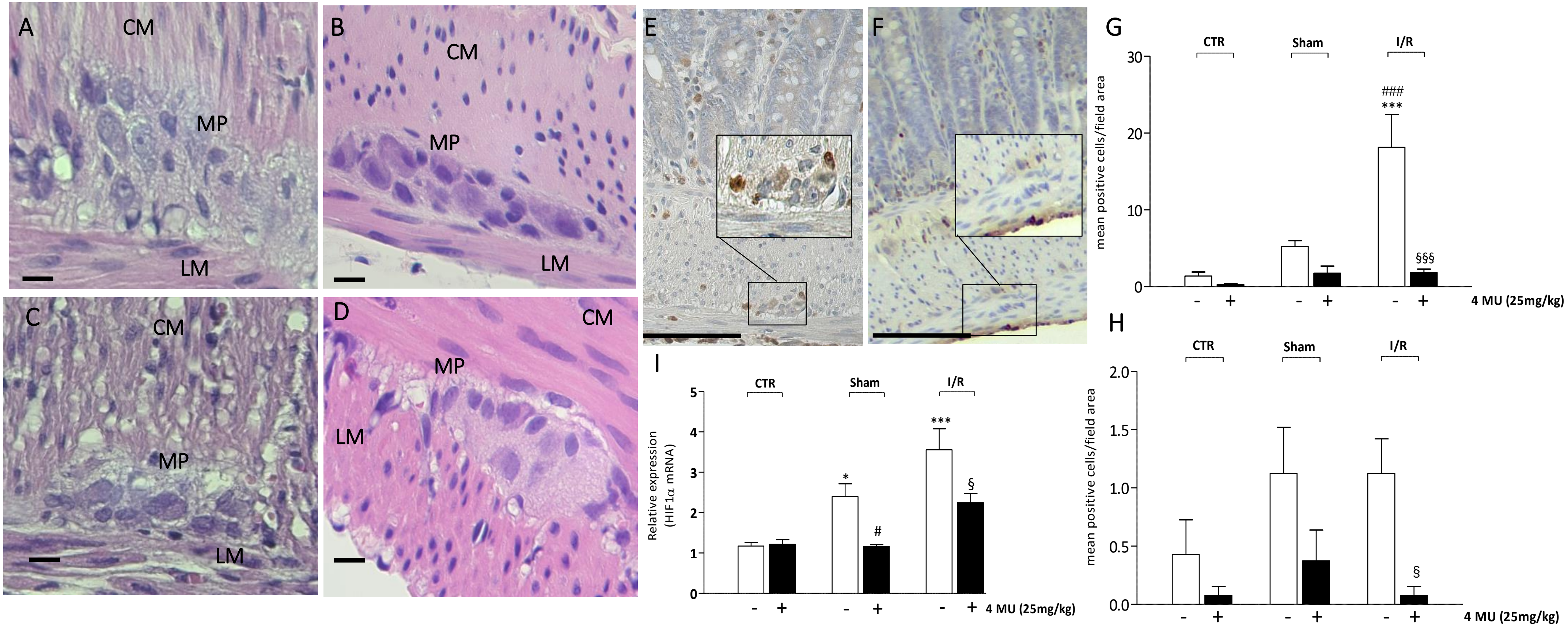

Figure S1: (A-D) smooth muscle cells in the circular (CM) and longitudinal (LM) layers and myenteric plexus (MP) of CTR (**A**), CTR 4-MU treated (**B**), I/R (**C**) and I/R 4-MU treated (**D**) rat small intestine (HE, original magnification 600x; bar: 0.01mm). (**C, D)**. **E-F**: MPO immunohistochemical staining of whole wall rat small intestine obtained from I/R and 4-MU treated I/R animals. Neutrophils are well marked (brown) and their count is easy for all the layers. Neutrophil infiltrate in the *muscularis propria* (**G**) and in myenteric ganglia (**H)** in the different experimental groups, expressed as number of neutrophils in the rat small intestine *muscularis propria*. Values are expressed as mean±SEM of neutrophil count. ***P<0.001 vs CTR, $^{###}$P<0.0001 vs Sham and §P<0,05 and §§§P<0.0001 vs I/R by one way ANOVA followed by Tukey's test. N=5 rats/group. **(I)** RT-PCR quantification of HIF1α in rat small intestine LMMPs in the different experimental groups. Values are expressed as means±SEM. N=5 rats/group. **P*<0.05, ****P*<0.001 vs CTR; $^{#}$P<0.05 vs Sham; §P<0.05 vs I/R by one way ANOVA followed by Tukey's test. Relative gene expression was determined by comparing $2^{-\Delta\Delta Ct}$ values normalized to β-actin.

Table S1

Frequency and amplitude of the spontaneous contractions in the rat small intestine longitudinal muscle recorded *in vitro* in the different experimental groups.

Experimental group	***Frequency (cicle/min)***	***Amplitude (g)***
**CTR**	17.79 ± 0.41	10.43 ± 0.92
**CTR 4-MU**	17.00 ± 0.56	10.47 ± 1.31
**sham**	16.75 ± 0.51	7.09 ± 0.74**
**sham 4-MU**	16.83 ± 0.46	7.13 ± 0.59**
**I/R**	18.45 ±0.51	7.56 ± 0,83*
**I/R 4-MU**	17.71 ± 0.45	6.67 ± 0.77**

Values are the mean ± SEM; n=5 rats/group. **P<0.001 and *P<0.05 vs CTR by one-way ANOVA with Tukey’s post hoc test.

Table S2

Number of myenteric neurons staining for HuC/D per ganglion area in rat small intestine longitudinal muscle myenteric plexus whole-mount preparations obtained from the different experimental groups.

Experimental group	***neuron number/ myenteric ganglion area (nm^2)***
**CTR**	250 ± 7.028
**CTR 4-MU**	238 ± 5.55
**sham**	229 ± 6.38
**sham 4-MU**	234 ± 5.29
**I/R**	238 ± 8.03
**I/R 4-MU**	253 ± 8.73

Values are the mean ± SEM; n=5 rats/group.

Table S3

Primary and secondary antisera and their respective dilutions used for immunohistochemistry (HC)

Antiserum	Dilution	Source	Host species
**Primary antisera**			
HABP, biotin	1:100	Hokudo (BC41)	--
HAS1	1:100	Bioss (bs-2946R)	Rabbit
HAS2	1:100	Santa Cruz (sc-34067)	Goat
HUC/D, biotin	1:100	Invitrogen (A-21272)	Mouse
VIP	1:200	Immunostar (20077)	Rabbit
nNOS	1:50	Santa Cruz (sc-648)	Rabbit
iNOS	1:50	Santa Cruz (sc-8310)	Rabbit
Substance P	1:200	Immunostar (20064)	Rabbit
ChAT	1:150	Abcam (ab70219)	Rabbit
**Secondary antisera and streptavidin complexes**			
FITC-conjugated streptavidin	1:200	Molecular Probes (SA1001)	---
Cy3-conjugated streptavidin	1:500	Amersham (PA43001)	---
Anti-rabbit Alexa Fluor 488	1:200	Molecular Probes (A21206)	Donkey
Anti-rabbit Alexa Fluor 555	1:500	Cell Signaling (4413)	Goat
Anti-mouse Alexa Fluor 488	1:200	Molecular Probes (A21202)	Donkey
Anti-goat Cy3	1:400	Jackson (705-165-147)	Donkey
F(ab')2 anti-mouse IgG (H+L) biotin	1:300	Caltag Laboratories (M35015)	Goat

Supplying companies: Abcam, Cambridge, UK; Amersham, GE Healthcare, Buckinghamshire, UK; Caltag Laboratories, Invitrogen, Burlingame, CA, USA; Dako, Glostrup, Demark; Jackson Immuno Research Laboratories, Inc., Baltimore, USA; Molecular Probes, Invitrogen, Carlsbad, CA, USA; Santa Cruz Biotechnology,CA, USA; Immunostar, Hudson, WI, USA; Bioss Antibodies, MA, USA; Hokudo, Nishi-ku, Sapporo, Japan.
